# Neural fields, spectral responses and lateral connections

**DOI:** 10.1016/j.neuroimage.2010.11.081

**Published:** 2011-03-01

**Authors:** D.A. Pinotsis, K.J. Friston

**Affiliations:** The Wellcome Trust Centre for Neuroimaging, University College London, Queen Square, London WC1N 3BG, UK

**Keywords:** Neural field theory, Spectral analysis, Turing instability, Dynamic causal modelling, Connectivity, Synaptic gain, Electrophysiology

## Abstract

This paper describes a neural field model for local (mesoscopic) dynamics on the cortical surface. Our focus is on sparse intrinsic connections that are characteristic of real cortical microcircuits. This sparsity is modelled with radial connectivity functions or kernels with non-central peaks. The ensuing analysis allows one to generate or predict spectral responses to known exogenous input or random fluctuations. Here, we characterise the effect of different connectivity architectures (the range, dispersion and propagation speed of intrinsic or lateral connections) and synaptic gains on spatiotemporal dynamics. Specifically, we look at spectral responses to random fluctuations and examine the ability of synaptic gain and connectivity parameters to induce Turing instabilities. We find that although the spatial deployment and speed of lateral connections can have a profound affect on the behaviour of spatial modes over different scales, only synaptic gain is capable of producing phase-transitions. We discuss the implications of these findings for the use of neural fields as generative models in dynamic causal modeling (DCM).

## Research highlights

►The modelling of sparse intrinsic connections with radial connectivity functions with distal peaks. ►A generative model of spectral responses to random fluctuations. ►The characterisation of the effect of different connectivity architectures (the range, dispersion and propagation speed of intrinsic or lateral connections) and synaptic gains on spatiotemporal dynamics of brain activity. ►We find that although the spatial deployment and speed of lateral connections can have a profound affect on the behaviour of spatial modes over different scales, only synaptic gain is capable of producing phase-transitions.

## Introduction

The spatiotemporal organisation of brain activity has been the focus of many empirical and theoretical studies, see e.g., (Bressler, 1984; Freeman, 1978; Jirsa et al., 1995; Spors and Grinvald, 2002; Valdes-Sosa, 2004). A dominant approach to spatiotemporal modelling of brain activity rests on neural field models: in the 70s Amari proposed an integrodifferential equation, which expressed neural activity as the response to input from neighbouring populations, assuming instantaneous postsynaptic processing (Amari, 1977). This approach has since been extended and is today known as neural field modeling (see (Deco et al., 2008), for a review of the relationship among mean-field, neural mass and field models).

Here, we employ neural field models to study how the spatial characteristics of intrinsic connections (connections confined to the cortical grey matter) affect the observed spectra of steady-state activity. In particular, we model spectral responses, due to interactions among coupled neural ensembles on the cortical surface, by assuming a precise topography of synaptic connections. Neural field models allow us to predict how the spatial organisation of coupling among populations is expressed in the dynamical repertoire of the system's response. Neural field models assume that the cortex is approximated by a homogeneous Euclidean manifold and neuronal activity at a given point is modelled by its average postsynaptic depolarisation. These models fall into the broad category of state-space models (Valdes-Sosa et al., 2009); a widely used class of such models are neural mass models, upon which various Dynamic Causal Models (DCMs) are based (Friston, 2006; Friston et al., 2003; Penny et al., 2004). However, neural field models differ substantially from neural mass models because the neural state is characterised by a mean depolarisation that is a function of space (in addition to time) and associated spatial parameters controlling the deployment and coupling of neuronal populations.

In this paper, we focus on one dimensional models and steady-state responses; namely, we assume that the activity of a neural ensemble occupying a local patch of cortex is perturbed around some steady-state due to random (exogenous) fluctuations. By transforming the state equation into the frequency domain, we characterise the response of the system by a transfer function. This furnishes a characterisation of frequency responses, in terms of intrinsic connectivity. The transfer function depends explicitly upon the spatial properties of the system, such as the spatial distribution of sources sending afferent (incoming) connections and the velocity of signal propagation. This is in contradistinction to the transfer function used in DCM for steady-state responses (Moran et al., 2009), which depends only upon lumped synaptic time constants and connection strengths.

We also consider the stability of the resulting neural field models in relation to the so-called Turing instability. In 1952 Turing suggested an explanation for the patterning of animal coats in terms of a reaction-diffusion process. He showed that an instability occurs during the system's transition from a regime where diffusion is absent to a regime where diffusion predominates. This instability leads to a spatially inhomogeneous state, which accounts for spontaneous pattern formation observed in a variety of processes. These ideas were first implemented in the context of neural field models by Wilson and Cowan (1972) and were used later by Ermentrout and Cowan (1979) to develop their theory of visual hallucinations. In the case of neural fields with axonal delays, the relevant analysis involves the notion of *dynamic* Turing instabilities. The first such instabilities were found by Bressloff (1996) and have been studied by other authors such as Hutt et al. (2003). Coombes and colleagues provide an excellent account of such phenomena in the context of neural fields (Coombes, 2005; Venkov et al., 2007).

Steady-state (or ongoing) activity spectra, associated with neural field equations, have been studied as models of the whole cortex (e.g. Jirsa, 2009). Robinson (2006) has developed a neurophysiologically informed field model of corticothalamic activity, which has proven successful in reproducing several properties of empirical EEG signals; such as the spectral peaks seen in various sleep states and seizure activity. In contrast to this work, the model used in our paper considers a local patch of cortex, as opposed to the global dynamics of the entire corticothalamic system. Furthermore, our focus is on the influence of intrinsic connectivity and the spatial characteristics of coupled neural populations at a mesoscopic level (i.e., in the order of a few millimetres) on the observed spectra, as opposed to considering the scalp EEG spectra measured from the whole brain. In particular, we will consider models that speak to the spatially discontinuous and patchy distribution of connections in the brain that are a hallmark of functional specialisation (Zeki, 1990). Functional specialisation demands that cells with common functional properties are grouped together. This architectural constraint necessitates both convergence and divergence of cortical connections. However, connections within and among cortical regions are not continuous but occur in patches or clusters and are mediated by axon collaterals that can extend for up to 5–7 mm (Angelucci and Bressloff, 2006). The existence of patchy connections in various cortical areas and various species has been well-established through extracellular tracer injection techniques, see Burkhalter and Bernardo (1989), Stettler et al. (2002) and Wallace and Bajwa (1991) for human, macaque and cat data respectively. Furthermore, it has been shown that such connections can have profound implications for neural field dynamics, see Baker and Cowan (2009).

This paper comprises two sections. In the first, we provide the basic theory behind our application of neural field models, with a special focus on spectral formulations and stability analyses. In the second section, we consider the implications of this theory for the influence of intrinsic connections on spectral activity, stability and pattern formation. Our interest here is on local (mesoscopic) dynamics that arise with sparse and patchy intrinsic or lateral connections. We model these with a slightly unusual connection kernel that has multiple non-central peaks. This allows us to manipulate both the range (modal distance) and dispersion of intrinsic connections. We then examine how changes in the range, dispersion, speed and synaptic gain of intrinsic connections affect spectral behaviour and stability. This kernel is unusual because the kernels in most neural field models decay with distance from the target location. However, this form of kernel does not capture the influence of horizontal connections from remote cortical patches that are characteristic of real intracortical connections. We conclude with a brief discussion of the applications of the models described in this paper, particularly their use as generative models and their inversion based on optical and other functional imaging data in the context of dynamic causal modelling.

## The neural field equation

### Transfer function

Neural field models are based upon complicated integrodifferential equations that usually preclude a full analytical treatment. Therefore, one usually resorts to suitable approximations to obtain solutions. The solutions of the corresponding integrodifferential equations include spatially and temporally periodic patterns beyond Turing instabilities; for example, localised regions of activity such as bumps and travelling waves, see (Pinto and Ermentrout, 2001) (Coombes et al, 2003), (Laing and Troy, 2003), (Hutt et al, 2003), (Atay and Hutt, 2006), (Laing and Chow, 2001) and (Rubin and Troy, 2004).

One well-known approach for obtaining solutions is to Fourier transform the neural field equation, therefore replacing the integrodifferential equation by a partial differential equation, under the assumption that the Fourier transform of the connectivity kernel (describing the spatial deployment of neuronal connections) is rational and well-behaved (Jirsa and Haken, 1997). Another approach to obtain solutions of the neural field equation is to perform a linear stability analysis: this assumes that the system is at a steady-state *v*_0_ and is perturbed by some external (exogenous) input *u*(*x*, *t*). If the input is stationary, then the ensuing activity corresponds to steady-state activity. If the input is manipulated experimentally (e.g., a stimulus), then the solutions yield induced or evoked responses. Here, we linearise around a steady-state to obtain an expression for the transfer function of the neural field equation. This allows us to express the system's spectral responses in terms of its key architectural parameters; namely its connectivity, post-synaptic gain and velocity of signal propagation. For notational clarity we will use lower case letters for time-varying quantities and upper case latter for their Fourier transform.

We start with the expected depolarisation *v*(*x*, *t*) at point *x* in the cortex, due to a presynaptic input from point *x*′,

(1) $$v(x,t)=\int_{-\infty }^{t}\sigma (t-t')\mu (x,t')\text{d}t'$$

where *μ*(*x*, *t*) is a spatial convolution of presynaptic input:

(2) $$\mu (x,t)=\int_{0}^{\infty }\int_{-\infty }^{\infty }\text{d}\left(\left|x'\right|,t'\right)f\left(v\left(x-x',t-t'\right)\right)\text{d}x'\text{d}t'+u(x,t)$$

and *σ*(*t*) models postsynaptic filtering of presynaptic inputs (firing)

(3) $$\sigma (t)=\{\begin{array}{cc} be^{-bt} & :t>0\\ 0 & \text{:}t\leq 0 \end{array}$$

The function *σ*(*t*) can be thought of as a Green's function of a differential operator, cf. (Amari, 1977). Here, *d*(|*x*|, *t*) = *κ*(|*x*|)*δ*(*t* − *ε*|*x*|) is a delayed version of a connectivity kernel *κ*(|*x*|) that accounts for axonal propagation delays. The conduction delay *ε* is the inverse of the speed with which signals propagate along connections*, b* is the average synaptic decay rate and *f*(*v*) is a post-synaptic firing rate function, which we assume to be a sigmoid.

Eqs. (1)–(3) are the precise analogues of the two main transformations associated with integrate and fire models (Jansen and Rit, 1995). Namely, Eq. (1) models the filtering of presynaptic input (firing rate) by the synaptic response function defined by Eq. (3) and transforms the presynaptic input into membrane depolarisation, *v*(*x*, *t*). Eq. (2) implements a weighted summation of activity delivered to neurons at point *x* from neurons at point *x*′ and transforms the average membrane depolarisation of the presynaptic population into firing rate, *μ*(*x*, *t*).Assuming that, as a result of constant external input *u*, the system is perturbed around a spatially homogeneous steady-state *v*_*0*,_ where,

(4) $$v_{0}=f(v_{0})\int_{-\infty }^{\infty }\kappa \left(\left|x\right|\right)\text{d}x+u$$

we can define the transfer function of the neural field by the following relation:

(5) $$D(k,\omega )=\frac{V(k,\omega )}{F(k,\omega )}$$

For simplicity, the transform of the synaptic convolution kernel has been omitted here under the adiabatic approximation that its time-constant is small in relation to fluctuations in voltage caused by exogenous input and neural field effects (i.e., we are assuming that *v*(*x*, *t*) ≈ *μ*(*x*, *t*)). Here, *V*(*k*, *ω*) is the Fourier–Laplace transform (a Fourier transform in the spatial domain and a Laplace transform in the time domain) of the depolarisation

(6) $$\begin{array}{c} V(k,\omega )=\text{FLT}(v(x,t))\\ =\int_{-\infty }^{\infty }dx\int_{0}^{\infty }dtv(x,t)e^{-ikx-\omega t} \end{array}$$

with ω∈C and *D*(*k*, *ω*) and *F*(*k*, *ω*) are defined similarly as the Fourier transforms of d(|*x*|, *t*) and *f*(*v*(*x*, *t*)) respectively. This means that we can characterise the spectral response of the system to any external input, in terms of the underlying connectivity kernel, propagation velocities and post-synaptic response function.

### The characteristic equation and spectral power

If we assume that fluctuations around the steady-state solution of the neural field Eq. (1) have the form $e^{\mathrm{\omega t}}e^{ik\cdot x}:\omega \in C\text{,}\;k\in R$, then these fluctuations represent decaying waves, with angular velocity Im(*ω*) and wavenumber *k* , propagating with phase velocity *υ* = Im(*ω*)/*k*. Substituting *v*(*x*, *t*) = *v*_0_ + *e*^*ωt*^*e*^*ik* ⋅ *x*^ into Eq. (1), we obtain (after expanding *f*(*v*) around *v*_0_),

(7) $$(\omega +1)e^{\mathrm{ikx}}e^{\mathrm{\omega t}}=\frac{g}{4}\int_{0}^{\infty }\int_{-\infty }^{\infty }d\left(\left|x^{\prime }\right|,t'\right)e^{ik(x-x^{\prime })}e^{\omega (t-t^{\prime })}dx'dt'$$

In this equation, we have rescaled time in terms of membrane time-constant and have used *f*′(*v*_0_) = *g*/4. In other words, we have approximated the firing rate function with a linear function of gain, *g*. The exponentials on both sides of Eq. (7) cancel and when we express Eq. (7) in terms of the Fourier-Laplace transform *D*(*k*, *ω*) of *d*(|*x*|, *t*), we obtain the *characteristic equation* of the linearised system *E*(*k*, *ω*)=0, where

(8) $$E(k,\omega )=\omega +1-\frac{g}{4}D(k,\omega )$$

Since the Fourier transform *D*(*k*, *ω*) is an explicit function of *k* and *ω*, it follows that the solution to Eq. (8) yields a dispersion relation *ω* = *ω*(*k*)*.* This relation connects the spatial and temporal properties of the perturbations to the steady-state. For a fixed value of the gain, the dispersion relation yields a curve in the complex *ω*-plane as a function of the wave number *k.* As we will see in Spectral responses, this curve entails the complex spectrum of the system and determines the stability of its responses. In particular, Turing instabilities occur if the spectrum crosses the imaginary axis from the left of the complex plane.

In the following, we use this relation to compute spectral responses of spatially extended neural populations. It should be emphasized that Eq. (8) is completely analogous to the characteristic equation associated with neural mass models (Moran et al., 2007). In both cases, the characteristic equations give the spectral responses of the corresponding neural system: in the case of linearised neural mass models, the spectral response is determined by the eigenspectrum (spectrum of eigenvalues) of the system's Jacobian, while for the neural field models considered here, this response is determined by the equivalent solution to the characteristic equation; i.e., the dispersion relation. In both cases, the frequency response of the system is analogous to the magnitude of the corresponding transfer function. Indeed, we have here,

(9) $$\left|\omega +1\right|\propto \left|D(k,\omega )\right|$$

This corresponds to the equation defining the frequency response for neural mass models. In both cases, Re(*ω*) expresses the rate in which the system returns to its resting state after a brief external perturbation and Im(*ω*) prescribes the frequency of these damped oscillations. However, there is an important difference between the neural mass approach and the approach used here: the characteristic Eq. (8) couples the spectral response of the system to the *spatial* properties of the system, such as the distribution of sources on the cortical sheet and delays due to finite propagation speeds. In neural mass models, the spatial properties of underlying neural populations are ignored and the spectrum is characterized only by rate constants.

In this paper, when we talk about spectra, we refer to the (complex) eigenspectrum that characterises the system's transfer function or mapping from inputs to responses. This is formally distinct from the spectral density associated with conventional time-series analysis, which we will refer to as power spectra or spectral power. However, spectral power can be derived easily from the transfer function, given the spectral power of the inputs: For example, under white noise input the power spectrum *S*(*k*, *ω*) for a spatial wavelength *λ* = 2*π*/*k* and temporal frequency *ω* is given by *S*(*k*, *ω*) = |*D*(*k*, *ω*)|^2^, where we have assumed *g* = 4. The integral over all *k* (spatial frequencies) yields the total power spectrum,

(10) $$S(\omega )={\left(2\pi \right)}^{-1}\int {\left|D(k,\omega )\right|}^{2}\text{d}k$$

Simulated EEG power spectra can be generated from Eq. (10) by replacing *D*(*k*, *ω*) with *D*(*k*, *ω*)*H*(*k*), where *H*(*k*) = *e*^− *k*/*k*_0_^ is a filter that accounts for volume conduction effects in the skull and scalp. This generally results in the attenuation of signal with wave-numbers *k* > *k*_0_, where *k*_0_ = 20 m^-1^ is a characteristic low-pass wave-number (Robinson, 2006).

### Stability

The dispersion relation *ω* = *ω*(*k*) determines the stability of the neural field equation. In particular, the steady-state is stable in the linear approximation if Re(*ω*) < 0, which is a condition assumed for steady-state responses. This implies that the spectra need to be on the left half of the complex plane (where the horizontal and the vertical axes depict the real and imaginary parts of *ω* respectively). Using a conformal mapping of the left hand complex plane to the interior of the unit disc, the above condition for stability can be recast as a condition that the spectra lie within the unit circle, cf. (Moran et al., 2007). Furthermore, Eq. (8) depends explicitly on the synaptic gain *g*; even if the system is initially at a fixed point changing the gain can render the system unstable. A similar phenomenon was observed numerically in (Moran et al., 2007) for neural mass models: increasing the gain led to an increase and broadening of the power and, at some point, a loss of stable band-pass properties. The mathematical description of this phenomenon appeals to the notion of Turing instability, which (for classical reaction-diffusion systems) is defined as an instability that emerges when the system leaves a spatially *homogeneous* resting state and enters an *inhomogeneous* state as a result of diffusion. This instability leads to spontaneous pattern formation, which (in two spatial dimensions) typically leads to stripes or hexagonal patterns. A treatment of such instabilities has been developed in the context of neural fields by (Wilson and Cowan, 1972). As we will see in Bifurcations, several instances of cortical dynamics could be described by Turing instabilities, for example pathological increases in synaptic gain associated with kindling and epilepsy.

In the context of neural field theory, the onset of dynamic Turing instability of the homogeneous steady state has been investigated and patterns emerging from this instability have been discussed in (Hutt et al., 2007), (Venkov et al, 2007), (Hutt et al., 2008), (Elvin et al., 2009). Also, the Turing instability analysis in layered 2D systems for neural fields with space-dependent delays is treated in (Coombes et al., 2007).

Assuming that axonal delays are negligible, it follows from Eq. (8) that for synaptic gain,

(11) $$g>\frac{4}{K(k)}$$

where *K*(*k*) denotes the Fourier transform of the connectivity kernel *κ*(|*x*|), the neural field model described by Eqs. (1)–(3), with *ε* = 0, is unstable. With axonal propagation delays, the analysis is a bit more complicated and leads to the notion of *dynamic* Turing instabilities. A Turing instability is induced by changing the value of a *control parameter*, such as the synaptic gain or the wave-number *k*_T_ corresponding to *ω*_T_. The instability arises when the spectrum crosses the imaginary axis for the first time. If *k*_*T*_ ≠ 0 then this wave-number corresponds to a travelling wave moving with velocity *ω*_T_/*k*_T_. If *k*_T_ = 0, then the Turing instability gives rise to another spatially uniform state. If *ω*_T_ has a non-zero imaginary part, then the Turing instability is called dynamic and is the solution of the neural field equation described by a limit cycle with frequency Im(*ω*_T_). In the next section, we consider a particular class of kernels describing intrinsic connectivity and the Turing instabilities induced by varying synaptic gain.

## Spectral responses

### Patchy connections

**Fig. 1:**
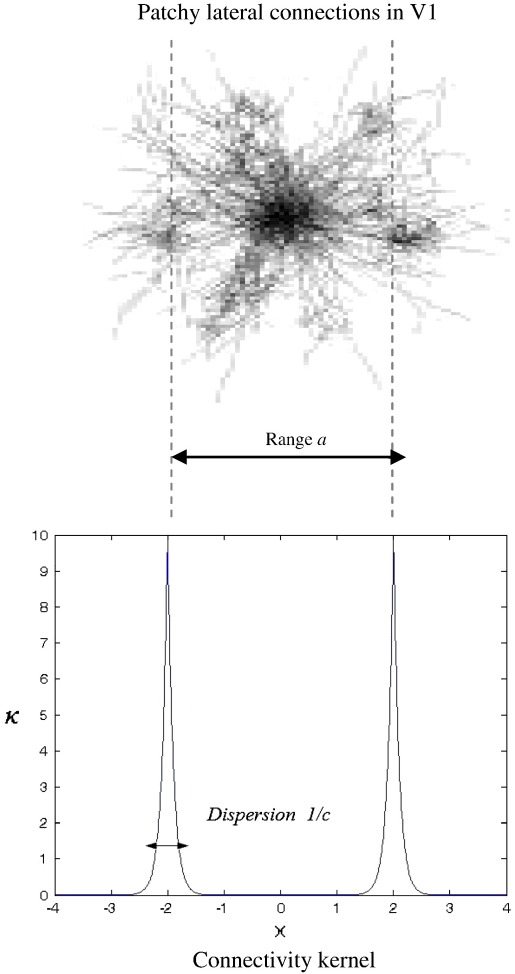
Connectivity kernel. Connectivity kernel describing the strength of intrinsic (lateral) connections within a neuronal field mode; see Eq. (12). The insert was modified from www.ini.uzh.ch/node/23776.

In the following, we consider a neural field model describing a system of interacting neural populations whose connections are not local but come from remote patches of the neural field; see Fig. 1. In other words, we are interested in modelling an ubiquitous aspect of horizontal connections in cortex; namely, their highly structured patchy or clustered organisation. These connectivity profiles have been studied most extensively in visual cortex (but see also Krubitzer and Kaas, 1990) and are generally thought the give rise to patches of functional segregation (e.g., ocular dominance, orientation selectivity *etc*.). See Angelucci and Bressloff (2006) for an excellent review. In short, neurons do not necessarily talk only to their immediate neighbours but send and receive connections preferentially with more remote populations who share the same functional selectivity; usually a millimetre or so away (e.g. Yoshioka et al., 1992). In a future work, we will consider kernels that have both remote and local component: Here, we focus on the dynamics that arise from non-local lateral interactions. We model the relevant connections with a connectivity kernel that has two peaks of height *c* located at distance *a* away from the target population (cf (Brackley and Turner, 2009) who consider inhomogeneous two point connections without delays as opposed to the homogeneous connections with conduction delays considered here),

(12) $$\kappa \left(\left|x\right|\right)=\frac{c}{e^{c\left|x-a\right|}}+\frac{c}{e^{c\left|x+a\right|}}$$

Kernels which are not peaked at the origin have been considered in the literature; see (Troy, 2008), (Hutt and Atay, 2005) and (Atay and Hutt, 2004). In particular, several studies focus on kernels that peak away from the origin, e.g. (Rinzel et al., 1998). However, the kernels considered here have the particular property that are symmetric around a point other than the origin (or comprise a sum of such terms). As explained in (Grindrod and Pinotsis, 2011), it is this property that leads to infinite-branched complex spectra discussed in below.

An example of a kernel of the form of Eq. (12) for *c* = 10 and *a* = 2 is given in Fig. 1.

The parameter *a* denotes the (modal) distance or range of neighbouring populations that contribute to the spectra of the target location. The parameter *c* describes the spatial extent of these contributions; namely, as *c* decreases, the bell-shaped curves of Fig. 1 become broader, which implies that the sources of afferent connections are more extensive or dispersed in space. On the other hand, in the limit *c* → *∞*, Eqs. (1)–(3) with connectivity defined by Eq. (12) describe a chain of neural masses coupled to each other over distances *a*. In short, Eq. (12) defines a class of connectivity kernels that account for nonlocal interactions between neighbouring neural populations that are parameterised in terms of the range and dispersion of lateral connections. Such kernels were introduced in Grindrod and Pinotsis (2011) and consist of a sum of terms that are symmetric around distal locations. They enable the parameterisation of interactions among distant populations as opposed to local interactions usually considered in the literature, see e.g. (Coombes et al., 2003; Venkov et al., 2007). Usually these connectivity kernels have a single peak at *x* = 0 making local behaviour the most dominant influence. The analysis of bimodal kernels involves the solution of transcendental equations and results in spectra with an infinite number of branches. Grindrod and Pinotsis consider several mathematical properties of the spectra for the closely related kernel

(13) $$\kappa \left(\left|x\right|\right)=\frac{r}{2e^{r\left|x-1\right|}}+\frac{r}{2e^{r\left|x+1\right|}}$$

and the relation between the neural field equation with axonal delays and differential-delay equations is demonstrated through the use of the so-called Lambert function. It should be noted that multibranched spectra of the sort obtained here include an infinite number of branches and are distinct from the multibranched spectra obtained in Nunez, (1995); a crucial difference being that the dispersion relation is transcendental as opposed to a sum of rational functions.

The kernel we consider in this paper, namely the function *κ*(|*x*|) defined by Eq. (12), is an explicit function of the distance of neighbouring populations producing observed spectral responses. This means we can study the spectral behaviour and stability of dynamics as a function of the range of “patchy” interactions. This is to be contrasted with the approach in (Grindrod and Pinotsis, 2011), where this distance was assumed to be fixed (and equal to unity). In short, the present approach exploits the explicit parameterisation of measured responses in terms of the spatial dispersion and range of intrinsic connections. Substituting Eq. (12) into the characteristic Eq. (8) and assuming connectivity has local support^1^ for *x* ∈ (− *a*, *a*), we obtain an expression coupling the spectral responses of the system with the relevant wave-numbers. This expression depends on the shape of the neuronal distributions, the distance between coupled populations and the conduction velocity, which are described by the variables *c, a* and *ε* respectively:

(14) $$\begin{array}{c} E(k,\omega ,a,\varepsilon )=\omega +1-\frac{g}{4}D(k,\omega )\\ D(k,\omega )=c[\frac{e^{-a\text{c}}c^{+}-e^{-\text{ε}a\text{ω}-2a\text{c}}(c^{+}\alpha +\beta )}{4k^{2}\pi^{2}+c^{+}c^{+}}+\frac{e^{-\text{ε}a\text{ω}}c^{-}\alpha -e^{-a\text{c}}c^{-}+e^{-\text{ε}a\text{ω}-a\text{c}}\beta }{4k^{2}\pi^{2}+c^{-}c^{-}}]\\ \alpha =\text{cos}(2ak\pi )\\ \beta =2k\pi \text{sin}(2ak\pi )\\ c^{+}=c+\varepsilon \omega \\ c^{-}=c-\varepsilon \omega \end{array}$$

In summary, the kernel defined by Eq. (12): (i) parameterises the spectral responses in terms of distance *a* between coupled populations; (ii) incorporates their spatial extent *c* and (iii) the propagation speed 1/*ε* of neuronal message passing. Equipped with this equation, we can now study the spectral power and stability of “patchy” neuronal interactions on the cortical sheet.

### Spectral power

**Fig. 2:**
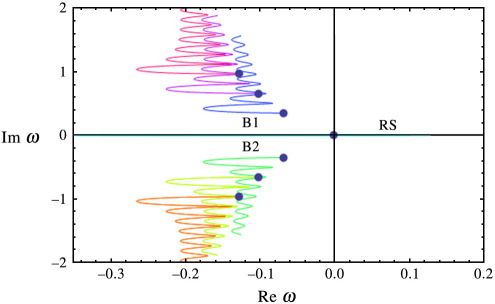
The spectrum obtained using the connectivity kernel (12). The spectrum *ω* of predicted steady-state responses using the bimodal connectivity scheme in Eq. (12). The spectrum is shown in the complex plane, where the imaginary part (vertical axis) determines the frequency of the response and the real part (horizontal axis) reflects the time course over which responses to perturbations decay (cf, the amplitude under white noise perturbations). The blue dots are the origin of each semi-branch at *k* = 0 (i.e. at the lowest spatial frequency). The semi-branches B1 and B2 are first order branches of multi-branched spectra associated with the neural field equation (for more details on spectra with an infinity of branches, see (Grindrod and Pinotsis)). Also, RS denotes the finite positive real part of the spectrum.

In Fig. 2, we depict the real versus the imaginary part of *ω* (the solution to Eq. (14) at different values of the wave-number). As with differential-delay equations, the spectrum of Eq. (1) with a connectivity kernel of the form of Eq. (12) has infinitely many branches, where each branch describes the dynamics of spatial modes of increasing wave-number.

Each coloured curve corresponds to a semi-branch of the frequency spectrum as explained in (Grindrod and Pinotsis, 2011). The appearance of multiple branches implies that for the same value of the real part of *ω*, depicted on the horizontal axis, there are multiple corresponding imaginary values. This can be seen by considering vertical lines, which intersect the coloured curves of Fig. 2 at multiple points. These imaginary values determine the observed frequencies, since the corresponding signals have a temporal profile of the form *e*^Re(*ω*)*t*^*e*^*i*Im(*ω*)*t*^. Furthermore, because the coefficients of *ω* in Eq. (8) are real, the spectrum has a symmetry around the horizontal axis; therefore, for every response with temporal profile *e*^Re(*ω*)*t*^*e*^*i*Im(*ω*)*t*^, there is another response with profile *e*^Re(*ω*)*t*^*e*^− *i*Im(*ω*)*t*^. It should be noted that, in contrast to the kernels considered in our earlier paper, the requirement of local support imposed here appears to have added a positive real part of finite length to the spectrum of the system (see the blue-green line “RS” on the positive real axis of Fig. 2, where RS denotes the finite positive real part of the spectrum). This part is present in all simulations below, although for clarity the positive real axis is omitted in subsequent figures. This is an interesting mathematical attribute of the current framework, which can account for the spontaneous appearance of decaying travelling waves of long wavelength, due to random perturbations and noise.

**Fig. 3:**
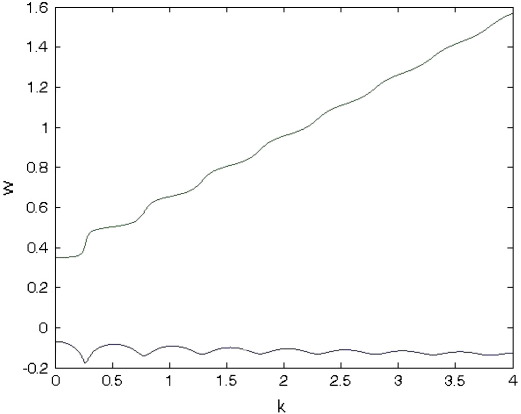
Real and imaginary parts of the spectrum. The blue and green curves are the real and imaginary parts respectively of a spectrum (the blue semi-branch in the previous figure) as a function of spatial wave-number (frequency).

The existence of multiple (in theory, infinite) branches in the spectra of the solution to the neural field equation depends crucially upon the choice of the kernel (12). This might seem counterintuitive to some, who might expect that the infinite degrees of freedom in any differential-delay equation (such as the neural field equation) would generate a multitude of branches, regardless of the choice of the kernel. However, for a large class of kernels commonly used in the literature, the spectrum is finite; for more details we refer the reader to (Grindrod and Pinotsis, 2011). We now focus on the structure of one of the semi-branches depicted in Fig. 2, namely the blue semi-branch, denoted by B1. By plotting the real and imaginary parts of this semi-branch against the spatial wave-number *k*, we obtain the results in Fig. 3.

The blue and green curves show the real and imaginary part of *ω* respectively. The real part stays negative as we increase *k*, therefore the spectrum is stable at all spatial frequencies. The real part fluctuates between (−0.2, 0) and for wave-numbers greater than *k* ∼ 3.5 it stabilises around −0.12. This implies that for *k* > 3.5 the oscillatory dynamics in these spatial modes will decay to zero at the same rate, regardless of the corresponding wave-number. Furthermore, the green curve reveals that increasing the wave-number progressively increases the frequency of steady-state oscillations; this is an intrinsic property of the connectivity kernel (15) and is not typical of all neural field systems with delays, see e.g. Jirsa (2009) where increasing *k* results in a saturation of temporal frequencies.

**Fig. 4:**
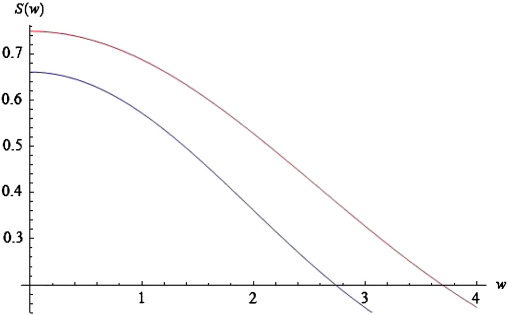
Log-power spectra for different values of connectivity range. Log-power spectra as a function of frequency, based on the solution to Eq. (14). Red curve for a connectivity range of a = 1 and blue curve for a = 1.3.

Substituting the expression for *D*(*k*, *ω*) in Eq. (14) into Eq. (10) furnishes the power spectrum for the neural field with connectivity defined by Eq. (12). We evaluated this integral numerically by adding terms for fixed values of *k* to produce the results in Fig. 4. Since the connectivity has local support in (− *a*, *a*) and assuming periodic boundary conditions*,* we can approximate this integral with

(15) $$S(\omega ,a,\varepsilon ,c)\approx \frac{1}{2a}\sum_{m=0}^{M}{\left|D(k_{m}=\pi m/a,\omega )\right|}^{2}$$

**Fig. 5:**
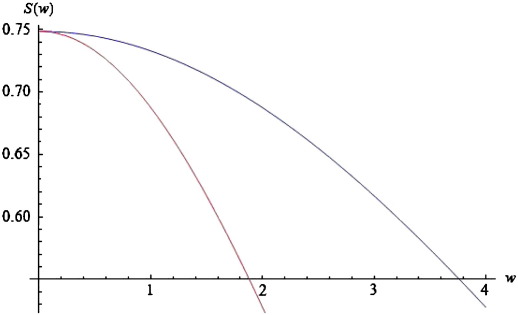
Log-power spectra for different values of conduction velocity. As for Fig. 4, but changing the conduction velocity from low (red curve: *ε* = 5) to higher (blue curve: *ε* = 2) values.

**Fig. 6:**
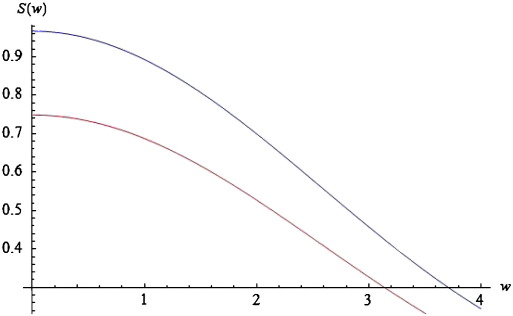
Log-power spectra for different values of dispersion. As for Fig. 4, but changing the spatial extent (dispersion) of afferent populations from *c* = 1 (red curve) to *c* = 2 (blue curve).

Choosing *ε* = 5 and *a* = *c* = 1, *g* = 4 and *M* = 40, we obtain the red curves in Figs. 4–6. These curves represent the log-power spectra (over spatial frequencies) as a function of temporal frequency *w* = Im(*ω*). These results depict a 1/*w* behaviour, which is typical for EEG power spectra that show scale-free regimes, in which the power falls in inverse proportion to frequency; (He et al., 2010; Jirsa, 2009; Kayser and Ermentrout, 2010; Robinson, 2006).

In Stability analysis, we will look at the stability properties of spectra, when changing various biophysical parameters. We conclude this subsection by illustrating the effects of these parameters on predicted power spectra and relate these effects to empirical observations. As the spatial separation of coupled neural populations increases, e.g., *a* = 1.3 (blue curve of Fig. 4), the total spectral power reduces and falls faster for higher frequencies, in a manner similar to local coherence functions observed in primate recordings, see Leopold et al. (2003). The opposite is the case as the velocity increases (see Fig. 5).

In this instance, higher frequencies are less damped. Furthermore, the effect of increasing velocity is apparent only for frequencies greater than *w* = 0.2. For lower frequencies, the blue and red curves coincide. These results accord with experimental results, where high frequencies are damped over shorter distances than lower frequencies. Finally, as *c* increases, the total power increases; and this effect appears to be stronger for lower frequencies. See the blue curve in Fig. 6. Again, these results are similar to the empirical observations of Leopold et al. (2003).

### Stability analysis

We can now investigate the influence of the biophysical parameters on the predicted spectra. Although it is straightforward to obtain corresponding results for higher order branches, for simplicity we will restrict ourselves to first order branches (corresponding to the rightmost semi-branches of Fig. 2). The following results were obtained using the numerical scheme of Grindrod and Pinotsis.

**Fig. 7:**
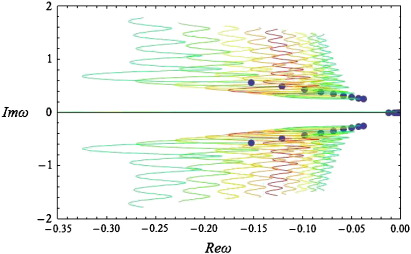
Complex spectra for various values of connectivity range. The spectrum *ω* of predicted steady-state responses using the bimodal connectivity scheme in Eq. (12). The spectrum is shown in the complex plane, where the imaginary and real parts of *ω* are depicted on the vertical and horizontal axes respectively (see also Fig. 2).Here c = 2, *ε*= 20 and we vary *a* between 0.6 and 1.4: Each coloured line corresponds to a first order branch for various values of *a*; the branch corresponding to *a* = 1 is depicted in red. Each blue dot depicts the corresponding origin of each branch.

Fig. 7 shows that as spatial separation (the range of intrinsic connections) *decreases* the spectra move to the left and tend to expand. This implies that the spectrum becomes more stable (decays more rapidly) and that higher imaginary values correspond to the same real part of *ω*. Hence, we would expect to see an increase in observed frequencies and reduced amplitude or increased dampening over frequencies (cf Figs. 4 and 5). Also, the real part is always present and its origin (zero order branch) moves away from (0,0) as separation decreases. In other words, uniform activity decays more quickly. Phenomenologically this is reminiscent of the desynchronisation seen in EEG activation studies (Brown and Marsden, 1999; Lemm et al., 2009; Pfurtscheller and Lopes da Silva, 1999), which entails a loss of high-amplitude slow fluctuations and an increase in faster dynamics. The current analysis suggests this sort of spectral behaviour rests could be reproduced by “shrinking” the range of lateral intrinsic interactions, e.g., through nonlinear synchronous gain mechanisms (Grossberg and Raizada, 2000; Li, 1998).

Clearly, we do not expect to see sustained oscillations at large separations: indeed, there is a critical value of *a*= 1.5, at which the multiple spectrum branch disappears. A similar phenomenon appears for *a*≤ 0.5 and may suggest our model cannot describe interactions over very short distances; this is consistent with our earlier observations, when we noted that kernels of the form of Eq. (12) are appropriate for describing non-local interactions as opposed to kernels which peak at the origin, such as the commonly used Mexican hat.

**Fig. 8:**
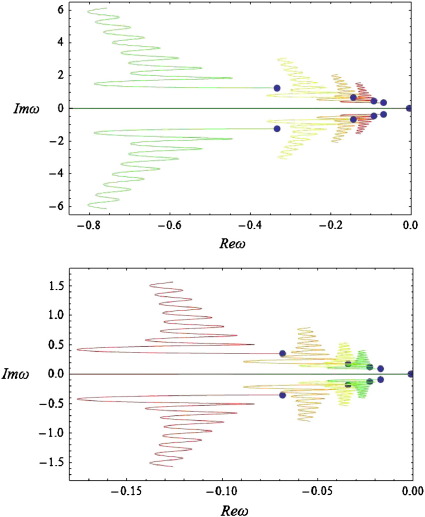
Complex spectra for various values of velocity. As for Fig. 7, but choosing c = 2, *a*= 1 and varying *ε* between one fourth and four times its original value *ε*= 20. The panels show a first order branch as the conduction velocity increases (above) or decreases (below); the branch corresponding to *ε*= 20 is depicted in red.

We next examined the effect of changing conduction velocities: The red branch in Fig. 8 is the same first order branch shown in Fig. 2. As the conduction velocity *increases* (*ε* decreases) the spectra move to the left and tend to expand, therefore we would expect to see higher frequencies, which are subject to more dampening. This is consistent with the “desynchronisation” observed when reducing the range of lateral interactions; in the sense that increasing the conduction velocities also reduces transmission delays between populations. However, the system appears to be more robust to changes in velocity, namely the infinitely branched spectrum is always present and does not disappear as was the case when varying *a* above. This means we expect to see sustained oscillations even for very small velocities.

**Fig. 9:**
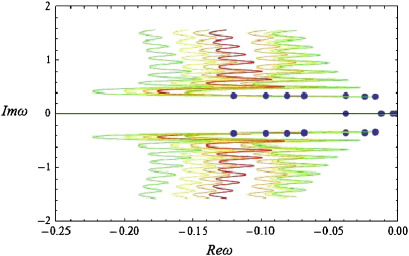
Complex spectra for various values of dispersion. As for Fig. 7, but choosing *ε*= 20, *a*= 1 and varying c between one fourth and four times its original value *c* = 2.

As the spatial extent (dispersion) of the afferent populations increases (*c* decreases), the system becomes more stable (Fig. 9). However, in contrast to the previous two cases, we do not see any change in the width of the spectrum and therefore would not expect to observe any change in frequencies. Also, the effect of changing *c* on higher frequencies appears to be smaller than the corresponding effect in lower frequencies (cf Fig. 6). It should be noted that although increasing *c* moves the real part of the spectrum towards zero, the spatial extent of the population does not affect the stability of the system qualitatively: In the limit *c* → *∞* (a chain of coupled neural masses), we recover the stable spectra of a system of differential-delay equations (Grindrod and Pinotsis, 2011). However, we show below that synaptic gain *g* can induce phase-transitions, which are manifest as spectra with cusps in the right hand side of the complex *ω* − plane. This is in accordance with the theoretical analysis of Turing instability, which establishes *g* as a control parameter (see Stability). Similar spectra were found when considering afferent populations at *x* = ± 1 and *x* = ± 2 (see Fig. 6 of Grindrod and Pinotsis).

### Bifurcations

**Fig. 10:**
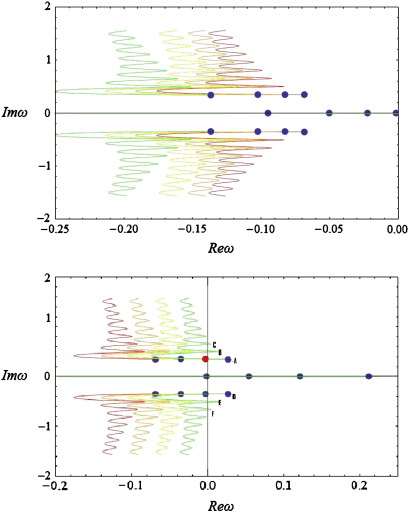
Complex spectra for various values of gain. As for Fig. 7, but choosing *ε*= 20, *a*= 1, *c* = 2 and varying g between one fourth and eight times its original value g = 4. The right and left panels show a first order branch as the gain decreases (above) or increases (below). The branch corresponding to *g* = 4 is depicted in red. A Hopf bifurcation occurs for *g* = 16 and temporal frequency *w* = 0.36 (red point in the right panel).

In the above simulations, we assumed *g =* 4 and obtained a series of spectra with stable complex parts (Re(*ω*) < 0 : ∀ Im(*ω*) ≠ 0) involving an infinite number of branches. Below, by changing the synaptic gain, we observe that the stability of the system alters as some of the branches cross the imaginary axis, leading to the appearance of limit cycles corresponding to waves on the cortex patch. In particular, varying *g* between one fourth and eight times its original value, we obtain the spectra shown in Fig. 10.

**Fig. 11:**
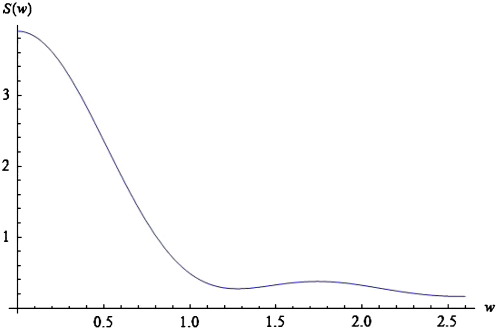
Log-power spectrum involving a peak. Log-power spectrum as a function of frequency (*w*), based on the solution to Eq. (14) with c = 2, *ε*= 5, *a* = 1 and *g* = 16. Note the peak at *w* = 1.7.

From Fig. 10 (above) we see that as *g decreases* the spectrum moves to the left (parallel to the real axis). Similarly, from the panel below, we conclude that for *g =* 16 (light green curve), the imaginary part of the spectrum reaches the imaginary axis for the first time, corresponding to a Hopf bifurcation at Im(*ω*_*T*_) = 0.36 (red point in Fig. 10—panel below). At this point self-sustaining oscillations (limit cycles) appear at a particular spatial scale, even in the absence of any perturbations. A local maximum appears in the corresponding power spectra, which ceases to exhibit a simple *1/w* form (Fig. 11). Empirical EEG power spectra with similar peaks may be associated with the emergence of these cortical waves as suggested in the pioneering work of Nunez, see (Nunez and Srinivasan, 2006) for a useful review.

Repeating the analysis of Spectral power, we find that changing the conduction velocity or the separation of cortical populations can have a profound effect on the dominant frequencies appearing in the simulated power spectra: The local maximum in Fig. 11 moves to the left and new local maxima appear as the delays increase or the connectivity range decreases, similar to the predictions of Nunez and Srinivasan (2006).

Further increases in gain drive the system into an unstable regime: for *g =* 32 (green line) three periodic solutions appear, with different frequencies with equal real parts of *ω* (points A, B and C of Fig. 10) along with their conjugate imaginary parts (points D, E and F). Clearly, in a linear system, these would not be observable because they would increase in amplitude as an exponential function of time. However, in real (nonlinear) neuronal systems the co-occurrence of different temporal frequencies at different spatial scales is characteristic of cortical dynamics in critical regimes, close to phase transitions: (Freeman, 2003; Freeman and Barrie, 2000). One might suppose that these sorts of dynamics arise during self-organised criticality (Bak et al., 1988; Kitzbichler et al., 2009) or indeed be evident in pathological increases in synaptic gain of the sort associated with kindling and epilepsy (Morimoto and Goddard, 1986; Wendling et al., 2002). It is interesting to note that most anti-epileptic drugs act through decreasing the sensitivity of population dynamics to afferent input by increasing inhibitory neurotransmission (Greenhill and Jones, 2010; Rigo et al., 2002). It also suggests that the cortical gain control is (literally) critical (Abbott et al., 1997). From a neuroscience perspective, the fact that changes in synaptic gain express themselves in a potentially qualitative fashion means that one may be able to infer synaptic gain parameters from observed spectral activity. We pursue this theme in the conclusion.

## Discussion

In this paper, we have described a neural field model for local (mesoscopic) dynamics on the cortical sheet. In general, neural fields are formulated in terms of integrodifferential equations with delays. Such equations have a rich repertoire of solutions including standing waves, bulk oscillations and travelling wave patterns that depend on the nature of the coupling kernel, see (Coombes, 2005), (Laing and Troy, 2003) and (Laing and Chow, 2001). Commonly used kernels, such as the Mexican hat render local interactions predominant and result in dispersion relations of a rational form as well as finite complex spectra. Here, we have introduced a class of kernels giving rise to dispersion relations of a transcendental form and spectra with an infinite number of branches. These kernels have non-central peaks that enable one to model (in an admittedly crude way) sparse intrinsic connections that are characteristic of real cortical microcircuits. The ensuing analysis allows one to generate or predict spectral responses to exogenous input or fluctuations. Crucially, we were able to characterise the effect of different connectivity architectures and synaptic gains on the ensuing spatiotemporal dynamics.

In principle, the fact that changes in the range or dispersion of intrinsic connections are expressed in terms of spectral behaviour means (Fig. 11) that these models can be used as generative (forward) models in dynamic causal modelling of empirical electrophysiological data. In other words, we can optimise the parameters of intrinsic connectivity and synaptic gain to best explain observed responses of an induced or evoked sort. Furthermore, the particular sensitivity to synaptic gain, in terms of critical slowing of various spatial modes and possible bifurcations (Fig. 10), means that inversion of these models may be particular sensitive to synaptic gain parameters (cf, Moran et al., 2009). Note that formulating the model in terms of a transfer function means that we have an implicit generative model of evoked or steady-state activity. The difference simply depends on whether the transfer functions are applied to known (experimental) deterministic inputs or random fluctuations with well-behaved statistics. In this paper, we have illustrated steady-state responses under white noise inputs, but the same transfer functions can also be applied to experimental inputs that are functions of peristimulus time.

The key advance that neural field models offer, over conventional neural mass models, is that they embody spatial parameters. This means one can infer the spatial parameters of cortical infrastructures generating electrophysiological signals (and infer changes in those parameters over different levels of an experimental factor). This rests on generating responses not just in time but also over spatial scales. Clearly, to exploit this sort of model, one would need to characterise the temporal dynamics of observed cortical responses over different spatial scales. In practice this would call for high-density recordings, probably at the epidural or intracortical level. However, data from optical imaging (Arieli et al., 1995; Zepeda et al., 2004) may also provide sufficient spatiotemporal resolution to support model inversion. The inversion of these models rests on a mapping from distributed cortical source activity to sensor data that can be specified accurately and preserves spatial information about the expression of different frequencies at different spatial scales (cf, the use of Wavelet decompositions; Schultze-Kraft, M., et al., 2010). One interesting issue here is that the curvature of the cortical surface may provide a rationale for the (difficult) inversion of forward models of non-invasive (e.g., scalp) data. This is because the spatial frequency “seen” by each electrode will be a function of the way the cortical manifold is embedded in the conduction volume. We will pursue this and related ideas in a subsequent paper.

We conclude with the usual qualifications about linear stability analyses, in relation to nonlinear systems, and the fact that we have only used a one-dimensional cortical sheet: Although we are working on two-dimensional formulations, we anticipate similar results, because of the rotational symmetry of the spatial kernels on which the model is based. We hope that the analyses presented in this paper are sufficient to show that neural field modelling has something interesting to say about spatiotemporal dynamics at both macroscopic and mesoscopic scales.

## Footnotes

1 A function has “local (compact) support” when it is non zero at a finite set of points of its domain.
